# Targeted proteomics reveal association between maternal biomarkers and child body weight in the first year of life

**DOI:** 10.1210/jendso/bvag052

**Published:** 2026-03-07

**Authors:** Noura Kabbani, Thomas Ebert, Holger Stepan, Massimiliano Lia, Matthias Blüher, Ronny Baber, Mandy Vogel, Ronald Biemann, Wieland Kiess, Anke Tönjes, Susanne Schrey-Petersen

**Affiliations:** Clinic and Polyclinic for Gynaecology, University of Leipzig Medical Center, Leipzig 04103, Germany; Leipzig Reproductive Health Research Center, Leipzig University, Leipzig 04103, Germany; Medical Department III–Endocrinology, Nephrology, Rheumatology, University of Leipzig Medical Center, Leipzig 04103, Germany; Leipzig Reproductive Health Research Center, Leipzig University, Leipzig 04103, Germany; Department of Obstetrics, University of Leipzig Medical Center, Leipzig 04103, Germany; Department of Obstetrics, University of Leipzig Medical Center, Leipzig 04103, Germany; Leipzig Reproductive Health Research Center, Leipzig University, Leipzig 04103, Germany; Helmholtz Institute for Metabolic, Obesity and Vascular Research of the Helmholtz Zentrum München, University of Leipzig and University Hospital Leipzig, Leipzig 04103, Germany; Leipzig Reproductive Health Research Center, Leipzig University, Leipzig 04103, Germany; Leipzig Medical Biobank, University of Leipzig Medical Center, Leipzig 04103, Germany; Institute of Laboratory Medicine, Clinical Chemistry and Molecular Diagnostics, University of Leipzig, Leipzig 04103, Germany; Leipzig Reproductive Health Research Center, Leipzig University, Leipzig 04103, Germany; LIFE Child, Hospital for Children and Adolescents, Department of Pediatrics, University of Leipzig Medical Center, Leipzig 04103, Germany; German Center for Child and Adolescent Health (DZKJ), Partner Site Leipzig/Dresden, Leipzig 04103, Germany; Institute of Laboratory Medicine, Clinical Chemistry and Molecular Diagnostics, University of Leipzig, Leipzig 04103, Germany; LIFE Child, Hospital for Children and Adolescents, Department of Pediatrics, University of Leipzig Medical Center, Leipzig 04103, Germany; German Center for Child and Adolescent Health (DZKJ), Partner Site Leipzig/Dresden, Leipzig 04103, Germany; Medical Department III–Endocrinology, Nephrology, Rheumatology, University of Leipzig Medical Center, Leipzig 04103, Germany; Department of Obstetrics, University of Leipzig Medical Center, Leipzig 04103, Germany

**Keywords:** biomarker, birth weight, fetal programming, ITGB2, olink proteomics, PON3

## Abstract

**Context:**

The intrauterine environment strongly influences children's health and development. Distinct cardiovascular biomarkers have been linked to birth weight and later weight gain, with correlations observed in maternal and umbilical cord serum.

**Objective:**

To describe (1) maternal cardiovascular biomarker patterns during the second and third trimesters and (2) potential associations between these biomarkers and offspring weight at birth and at 1 year of age.

**Design:**

Within the LIFE Child Study, serum samples from 86 healthy mothers at 24 and 36 gestational weeks and cord blood at birth were analyzed using the Olink® Target 96 Cardiovascular III panel. Statistical analyses (Wilcoxon test, Spearman correlation, multivariate regression) were performed in R.

**Setting:**

Community-based cohort, Leipzig, Germany.

**Patients or Other Participants:**

Eighty-six mother–child pairs from the LIFE Child cohort. Mothers had no pregnancy complications, and all newborns had birth weights between 2500 and 4500 g.

**Main Outcome Measure:**

Offspring body weight at 1 year of age.

**Results:**

Of 92 maternal serum biomarkers, 88 were detectable. Seventy biomarkers increased significantly from 24 to 36 weeks (*P* < .004). Several biomarkers measured at the 36th gestational week correlated with birth weight and 1-year weight. After adjustment for maternal age, body mass index, and offspring sex, no associations remained with birth weight. However, maternal paraoxonase 3 (PON3) [*P* = .037, 95% confidence interval (CI): −0.52, −0.02] and integrin subunit β 2 (ITGB2) (*P* = .038, 95% CI: 0.04, 1.12) were significantly associated with child weight at 1 year.

**Conclusion:**

In our cohort, maternal PON3 and ITGB2 were independently associated with early postnatal growth, potentially implicating these biomarkers in fetal programming.

The association between birth weight and adverse neonatal outcomes has been investigated in detail in previous studies [1, 2]. Both small-for-gestational-age (SGA) and large-for-gestational-age (LGA) neonates have been found to be at higher risk of cardiovascular disease in adulthood [3]. SGA birth weight is defined as birth weight below the 10th percentile of given reference ranges, while LGA can be defined as birth weight above 10th percentile of given reference ranges [4]. Indeed, as Barker's hypothesis proposes, fetal programming of metabolic processes can shape the trajectory of offspring health well into adulthood [2]. Furthermore, SGA fetal weight is often considered a surrogate marker for increased risk of intrauterine fetal demise and adverse perinatal outcomes, highlighting its prognostic value for perinatal morbidity and mortality. Predictors of adverse neonatal outcomes, particularly those based around birth weight, have been the subject of much research in recent years [5-9]. To date, SGA birth weight as predicted through ultrasound measurements is 1 of the most common used surrogate parameters for adverse neonatal outcome [10, 11].

Importantly, low birth weight has additionally been implied in higher cardiovascular risk in adulthood [12, 13]. Moreover, weight gain in early childhood has been linked to adult health outcomes. For example, weight gain in the first years of life, especially before the age of 2, has been shown to be associated with cardiovascular health in later life [14]. For these reasons, birth weight below the 10th percentile (ie, SGA) may be inadequate as the sole parameter to characterize the neonates' health risk in later life. To better understand the pathomechanisms underlying abnormal child growth, it is crucial to first investigate these processes in healthy children. However, data on this topic remain limited, underscoring the need for further analysis of children born with appropriate-for-gestational-age birth weights.

Several maternal biomarkers have been presented as predictors of pregnancy complications for both mother and child [15-17]. For example, maternal CCL20 has been identified as a potential predictive and diagnostic marker of preeclampsia [18], while the adipokines resistin and chemerin have been proposed to be associated with the development of gestational diabetes in expectant mothers [16, 19]. While maternal biomarkers, most famously leptin, have been linked to birth weight, the evidence surrounding biomarkers predicting child weight gain in early childhood is sparse [20].

Mid- and late pregnancy are critical periods for fetal growth and maternal cardiovascular adaptation, and maternal cardiovascular biomarkers during this time may provide insight not only into birth outcomes but also into early childhood weight gain and growth trajectories along standardized percentiles. However, the gestational period during which maternal biomarkers have the strongest predictive value for later child health remains unclear. While most studies have focused on these biomarkers in the context of obstetric complications or birth weight, their association with postnatal child growth and cardiovascular risk remains largely unexplored.

Such biomarkers, which would help to predict child weight development in early childhood, remain to be identified. Thus, it is indispensable to study the maternal biomolecular mechanisms that influence intrauterine development and early as well as later growth.

The aims of this study were to describe (1) the patterns of maternal cardiovascular biomarkers during the second and third trimesters and (2) possible associations between these maternal biomarkers and the children's body weight at birth as well as at 1 year of age.

## Materials and methods

### Study design and subjects

This was a subgroup analysis of the LIFE Child study, a population-based longitudinal observational study [21]. This large cohort study (>5500 children) aims to assess the effects of various genetic, lifestyle, and environmental factors on child health and development by various examinations from pregnancy up to adolescence [21]. As part of this study, serum samples of healthy pregnant women with a nonanomalous fetus were collected at the 24th and 36th week of gestation in order to measure the levels of various cardiovascular biomarkers. Child growth development was assessed by standardized pediatric examinations, including the measurement of height, weight, and head circumference at birth and 1 year of age. Additionally, clinical data of the course of pregnancy as well as on birth outcome were recorded.

For this analysis, inclusion criteria were as follows: women between 18 and 41 years of age without any known underlying cardiovascular disease and with healthy, uncomplicated singleton pregnancies, as well as uncomplicated births between the 37th and 40th gestational week. Women with diabetes or any proven glucose intolerance were excluded. Inclusion criteria for offspring included appropriate-for-gestational-age birth weight (≥third percentile, weight range in our cohort 2500-4500 g), the absence of syndromal or chromosomal diseases, and a 10-minute APGAR score ≥ 8. Conversely, exclusion criteria for pregnant women included chronic, chromosomal or syndromic diseases, pregnancy complications, such as gestational diabetes and preeclampsia, as well as preterm births (<37 weeks’ gestation).

Maternal clinical and anthropometric measurements recorded throughout pregnancy included age, prepregnancy weight, and gestational weight gain. In all children, anthropometric measurements were taken at birth as well as 1 year of age, including body weight and weight SD score (SDS; based on Kromeyer–Hauschild SD scores [22]) adjusted for gestational age and/or percentile. Weight gain at 1 year of age was defined as weight at 1 year of age minus birth weight.

Serum samples taken at the 36th week of gestation were available for all 86 mothers, while additional serum samples taken at the 24th week of gestation were available for 60 out of 86 mothers in our cohort.

The primary outcome was birth weight, as well as children’s body weight at 1 year of age, standardized and adjusted for gestational age at birth.

### Assays and laboratory measurements

All serum samples were managed by the Leipzig Medical Biobank team according to standard operating procedures. The predetermined fasting times before serum sampling for subjects were 12 hours in the second trimester and at least 4 hours in the third trimester to avoid prolonged fasting times during late pregnancy. Samples drawn from pregnant women were processed within 180 minutes after blood collection. All samples were stored at −80 °C [2D-barcoded cryotubes (Azenta)] or at temperatures <150 °C in straws (Cryo Bio Systems IMV). Maternal cardiovascular biomarkers were quantified using Olink Target 96 cardiovascular III panels (Olink® Proteomics AB, Uppsala, Sweden, AB_3739858) based on the proximity extension assay technique. This panel comprises 92 different cardiovascular biomarkers [23]. The complete list of proteins included in the Olink panel is provided in Table S1 [24].

The high-throughput proximity extension assay technique utilizes immunoassays with oligonucleotide-labeled antibodies followed by real-time PCR for simultaneous quantification of target proteins with high specificity and scalability [25]. After intensity normalization (Olink NPX Signature 1.4.0.1, Olink Proteomics), the relative concentration of measured serum proteins is expressed as normalized protein expression values (NPX) for each protein with relative quantification using the log2-scale (ie, 1 NPX difference equaling a 2-fold change in protein concentration). All samples were randomized across plates, and interassay coefficients of variance (CV) are based on control samples (pooled plasma samples) included on each plate. The average intra-assay CV was 5% (reference <15%), while the average interassay CV was 7% (reference <25%) (data provided by our in-house Olink Proteomics unit).

### Statistical analysis

The statistical software environment R [26] (Version 4.2.2) was used for data analysis, regression analysis (rms-package), Spearman's rank correlations (Hmisc-package), and drafting of graphics (ggplot2-package and pheatmap package).

The Wilcoxon signed-rank test was used to longitudinally compare biomarker levels between the 24th and 36th week of gestation.

Spearman's rank correlation was used to screen between maternal serum parameters (at the 36th week of gestation) and the primary outcome (ie, birth weight and child's weight at 1 year). Each serum parameter with statistically significant correlation (*P* < .05) with birth weight was analyzed with multivariable regression in order to assess if its association with the outcome remained significant after adjustment for maternal body mass index (BMI), maternal age, and child sex; smoking and alcohol consumption were not included due to incomplete self-reported data. The same approach was used to examine associations between maternal serum parameters and child's weight at 1 year.

## Results

From the LIFE Child cohort, 87 mothers matched the inclusion criteria for this study. We excluded 1 case due to missing children’s body weight at 1 year, leading to a final study cohort comprising 86 mothers and their 86 children.

Cohort characteristics are summarized in Tables 1 and 2 for mothers and their children, respectively.

**Table 1:** Study cohort: maternal characteristics of the entire study cohort (n = 86)

Parameter	Unit	Mean	SD
Age	Years	30.4	±4.61
Body weight (prepregnancy)	kg	66.6	±12.62
Body weight (end of pregnancy	kg	80.9	±13.08
BMI (before pregnancy)	kg/m^2^	23.8	±4.49
BMI (end of pregnancy)	kg/m^2^	29.1	±4.75
Duration of pregnancy	Weeks	39.9	±1.23

Abbreviations: BMI, body mass index.

**Table 2:** Study cohort: offspring characteristics (n = 86: female = 36, male = 50)

Parameter	Unit	Birth		1 year of age	
		Mean	SD	Mean	SD
Gestational age	*z*-score	0.00	±0.02	—	—
Age	Years	—	—	1.0	±0.08
Body weight	G	3464	±396.41	9447	±1049.26
Body weight SDS	—	0.2	±0.80	−0.2	±0.75
Body length	cm	49.9	±1.86	75.0	±2.86
Head circumference	cm	35.2	±1.44	46.1	±1.67

Abbreviations: SDS, SD score.

Of the 92 biomarkers measured in the Olink panel, 4 biomarkers showed NPX values below the limit of detection. Therefore, a total of 88 biomarkers could be analyzed for the purposes of our study. The excluded biomarkers were CD236 (Ep-CAM), N-terminal pro brain natriuretic peptide, pulmonary surfactant-associated protein D, and spondin-1.

### Maternal serum biomarker levels between the 24th and 36th week of gestation

**Figure 1:**
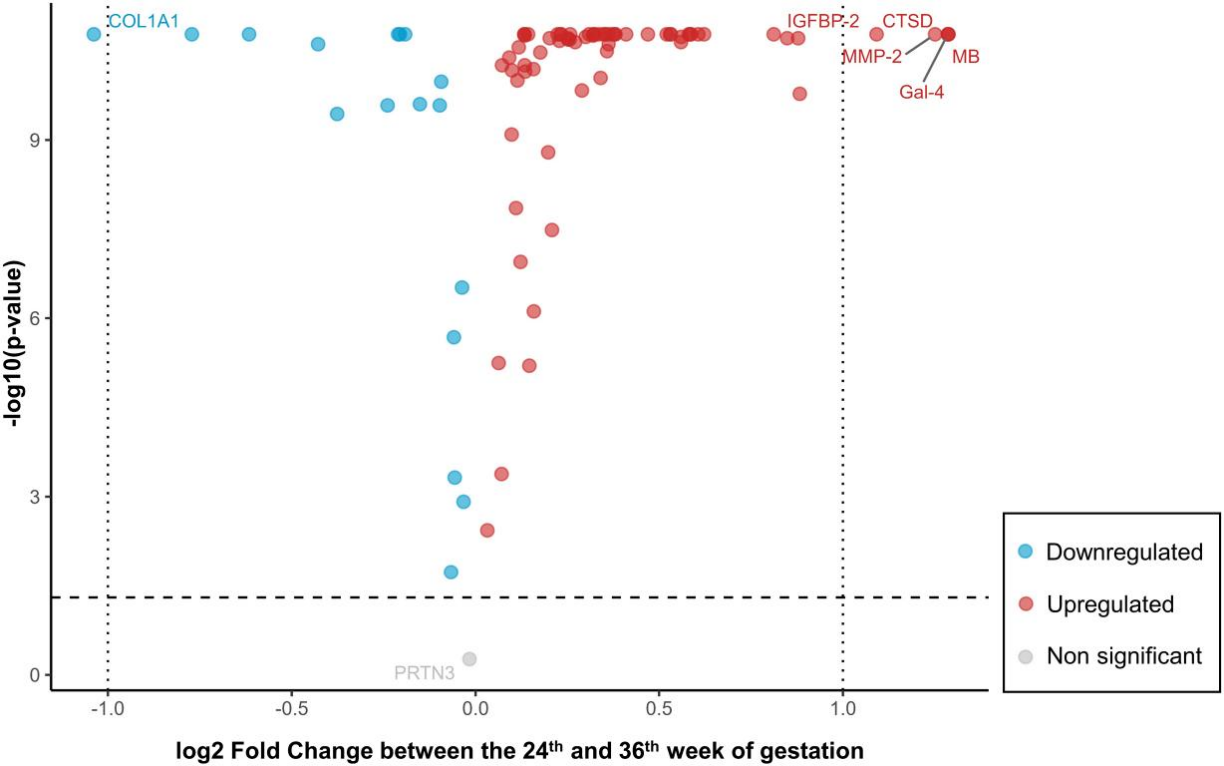
Volcano plot depicting changes in biomarker NPX values in maternal serum between the 24th and 36th week of gestation. The x-axis shows log2-fold changes in NPX levels, while the y-axis displays the log10 *P*-values, indicating statistical significance. Colored points represent significantly up- or downregulated biomarkers, whereas grey points indicate nonsignificant changes. Biomarker abbreviations as indicated in Table S1 [24]. Abbreviations: GW, gestational week; NPX, normal protein expression.

Significant differences in circulating levels of maternal biomarkers were observed between the 24th and 36th week of gestation in 87 out of the 88 biomarkers with values above the limit of detection. Thus, about 70 markers increased significantly from the second until the third trimester with cathepsin D, myoglobin, and IGF-binding protein 2 showing over 2-fold increase (all *P* < .05) (Fig. 1). Conversely, 17 biomarkers decreased significantly in the third trimester with most pronounced effects found for collagen α-1(I) chain, which showed a decrease of approximately 7-fold (all *P* < .05). In contrast, myeloblastin (proteinase 3) was the only biomarker that did not show any changes from the second until the third trimester. Biomarker dynamics between the second and third trimesters for all measured biomarkers can be found in the Supplemental Materials [24].

### Association between maternal serum biomarkers at the 36th week of gestation and child birth weight parameters

Significant correlations were found between 4 maternal serum biomarkers at the 36th week of gestation and birth weight. Aminopeptidase N, cadherin-5, carboxypeptidase A1, and platelet-derived growth factor subunit A all correlated significantly and negatively with birth weight. All significant correlations between maternal serum biomarkers and child birth weight parameters are shown in Table 3. A visual representation of these correlations is available in Supplemental Fig. S1 [24].

**Table 3:** Significant correlations between maternal serum biomarkers and child birth weight

Biomarker	Birth weight		Birth weight SDS		Birth weight SDS adj. for GA	
	r	*P*	r	*P*	r	*P*
AP-N	−0.265	.075	−0.269	.071	−0.342	.**020**
CDH5	−0.227	.129	−0.217	.148	−0.321	.**029**
CPA1	−0.231	.122	−0.265	.075	−0.300	.**043**
PDGFA	−0.278	.061	−0.266	.074	−0.307	.**038**

Correlations were calculated between maternal serum biomarker normal protein expression values and child birth weight SDS adjusted for gestational age. No additional covariate adjustment was applied in these correlation analyses. Significant values are printed in bold text.

Abbreviations: AP-N, aminopeptidase N; CDH5, cadherin-5; CPA1, carboxypeptidase A1; GA, gestational age; PDGFA, platelet-derived growth factor subunit A; SDS, Kromeyer–Hauschild SD score.

**Figure 2:**
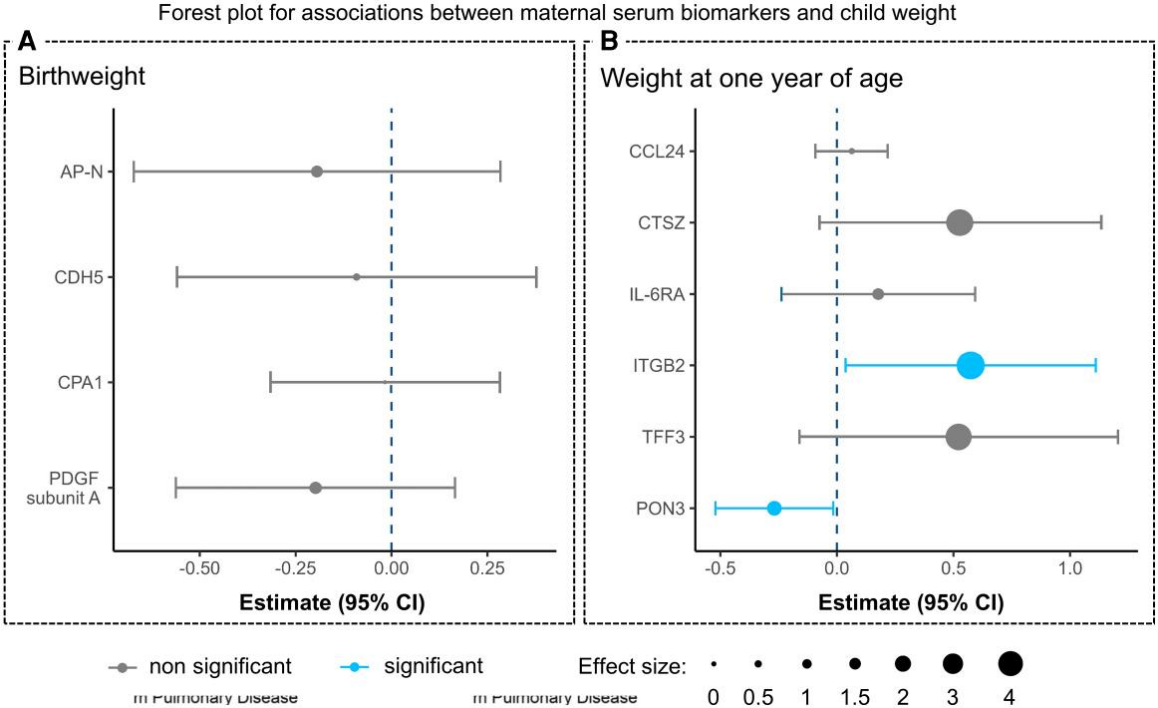
(A) Forest plot for associations between maternal serum biomarkers and child birth weight, adjusted for maternal body mass index and age, as well as child sex. Level of significance indicated by color code. Strength of the observed associations is provided by standardized β coefficients with 95% confidence intervals and is further indicated by circle diameter. (B) Forest plot for associations between maternal serum biomarkers and child body weight at 1 year of age, adjusted for maternal body mass index and age, as well as child sex. Level of significance indicated by color code. Strength of the observed associations is provided by standardized β coefficients with 95% confidence intervals and is further indicated by circle diameter. Biomarker abbreviations as indicated in Table S1 [24].

However, maternal serum biomarkers with a significant correlation to birth weight did not retain statistical significance after adjustment for maternal age, maternal BMI, and offspring sex (Fig. 2A).

### Association between maternal serum biomarkers at the 36th week of gestation and child weight parameters at 1 year of age

Six maternal serum biomarkers in the 36th gestational week showed significant correlations with child weight at 1 year of age. Interestingly, 10 biomarkers correlated positively and significantly with weight gain at 1 year of age, with C-C motif chemokine 24, cathepsin Z, and paroxonase 3 (PON3) showing the largest absolute value for correlation coefficients. Table 4 shows all biomarkers with significant correlations.

**Table 4:** Significant correlations between maternal serum biomarkers and child weight at 1 year of age

Biomarker	Body weight		Body weight SDS		Body weight SDS adj. for GA		Weight gain (weight at 1 year – birth weight)	
	r	*P*	r	*P*	r	*P*	r	*P*
AP-N	**−0**.**292**	.**049**	−0.219	.143	−0.236	.114	−0.196	.192
AXL	0.265	.075	**0**.**312**	.**035**	0.286	.054	0.241	.107
CCL16	**0**.**347**	.**018**	**0**.**307**	.**038**	0.281	.059	**0**.**300**	.**043**
CCL24	0.291	.050	0.288	.052	**0**.**292**	.**049**	**0**.**442**	.**002**
CNTN1	**−0**.**320**	.**030**	−0.155	.303	−0.196	.192	**−0**.**293**	.**048**
CTSZ	**0**.**400**	.**006**	**0**.**367**	.**012**	**0**.**379**	.**009**	**0**.**420**	.**004**
IL-6RA	**0**.**326**	.**027**	**0**.**352**	.**017**	**0**.**328**	.**026**	**0**.**385**	.**008**
ITGB2	0.192	.201	**0**.**318**	.**031**	**0**.**313**	.**034**	0.240	.108
OPN	0.238	.112	0.256	.087	0.248	.097	**0**.**322**	.**029**
PLC	0.281	.058	0.277	.063	0.266	.074	**0**.**319**	.**031**
PON3	**−0**.**451**	.**002**	**−0**.**317**	.**032**	**−0**.**340**	.**021**	**−0**.**473**	.**001**
RARRES2	0.251	.092	0.196	.192	0.177	.238	**0**.**298**	.**044**
TFF3	**0**.**388**	.**008**	**0**.**461**	.**001**	**0**.**454**	.**002**	**0**.**408**	.**005**

Correlations were calculated between maternal serum biomarker normal protein expression values and child body weight SDS adjusted for gestational age. No additional covariate adjustment was applied in these correlation analyses. Significant values are printed in bold text.

Abbreviations: AP-N, aminopeptidase N; AXL, AXL receptor tyrosine kinase; CCL16, C-C motif chemokine 16; CCL24, C-C motif chemokine 24; CNTN1, Contactin-1; CTSZ, cathepsin Z; GA, gestational age; IL-6RA, interleukin-6 receptor alpha; ITGB2, integrin beta-2; OPN, osteopontin; PLC, phospholipase C; PON3, paroxonase 3; RARRES2, chemerin; SDS, Kromeyer–Hauschild SD score; TFF3, trefoil factor 3.

Both ITGB2 (*P* = .038, 95% confidence interval: 0.04, 1.12) and PON3 (*P* = .037, 95% confidence interval: −0.52, −0.02) retained a statistically significant association with body weight at 1 year after adjustment for maternal age, maternal BMI, and offspring sex in multivariable regression (Fig. 2B). In contrast, the other biomarkers (cathepsin Z, T, IL-6 receptor α, and C-C motif chemokine 24) did not show any significant association after statistical adjustment.

## Discussion

### Principal findings

In our study, we show that maternal serum concentrations of most biomarkers analyzed by our targeted proteomics analysis increase during the end of pregnancy. Second, the maternal biomarkers PON3 and ITGB2 measured during pregnancy show significant associations with weight development in infants born with an appropriate-for-gestational-age birth weight during the first year of childhood. These associations remained even after adjustment for maternal age and BMI, as well as offspring sex.

### Results in the context of what is known

#### Maternal biomarker expression throughout pregnancy

Several studies have investigated changes in biomarker levels throughout pregnancy. In line with our findings, previous studies have reported increasing levels of resistin, chemerin, and growth differentiation factor 15 during gestation [19, 27-31]. These studies proposed a pathogenic link to maternal insulin resistance as well as glucose tolerance.

Interestingly, we found that ITGB2 in maternal serum, showing significant associations with weight SDS at 1 year of age adjusted for GA, was significantly increased in the 36th gestational week. In contrast, PON3, which similarly showed a significant association with weight SDS at 1 year of age adjusted for GA, decreased significantly in the third trimester. Our analysis revealed serum levels of most of the measured biomarkers increased from the second to the third trimester. This could be the result of known increased cardiometabolic stress for mothers toward the end of pregnancy. It could further point to their possible involvement in biological processes related to pregnancy progression and fetal development.

#### Maternal biomarkers at birth and child birth weight

The absence of significant associations between maternal serum biomarkers and offspring birth weight after adjustment for maternal BMI, maternal age, and offspring sex suggests that birth weight is not strongly connected with maternal cardiovascular state. However, it is known that a lower birth weight is linked to cardiovascular risk as well as cardiovascular disease later on in life [3, 32, 33].

Studies have shown that birth weight is associated with fetal genotype, with fetal genotype influencing up to 15% of a child’s birth weight [32]. Previous studies suggest that fetal genotype and maternal genetic factors are major contributors to offspring cardiovascular risk [32, 33].

Taken together, these findings suggest that genetic and fetal determinants may play a more prominent role in regulating birth weight than maternal cardiovascular biomarker profiles measured at birth, potentially explaining the lack of associations observed in the present study.

#### Maternal biomarkers at birth and child weight at 1 year of age

Of the biomarkers included in our analysis, 2 showed significant associations with child weight at 1 year of age after adjustment for maternal age, BMI, and child sex.

PON3 is part of the paraoxonase gene family comprising PON1, PON2, and PON3 and is primarily secreted by the liver [34, 35]. Through its antioxidative and anti-inflammatory properties, PON3 has been found to play a protective role in metabolic diseases such as obesity as well as steatotic liver disease [35, 36]. It has been suggested that PON3 can modulate mitochondrial function and therefore oxidative stress, as well as prevent apoptosis [37]. Similarly, PON3 has been found to influence the metabolism of lipids as well as bile acids [37]. In animal models, knockout mice showed increased body weight and a higher susceptibility to obesity and atherosclerosis [38].

In the context of pregnancy, elevated PON3 levels are associated with increased oocyte quality as well as higher fertilization rates in assisted reproductive technologies [39]. Although previous studies reported higher cord blood PON3 levels in term infants compared with preterm infants [40], our study found that maternal circulating PON3 levels at 36 weeks of gestation were decreased.

Encoded by the *ITGB2* gene, the protein ITGB2 is crucial for the function of the immune system [41]. It enables leukocyte adhesion and migration and therefore the modulation of immune responses [41]. Deficiencies in ITGB2 expression can lead to immunological disorders, such as leukocyte adhesion deficiency type 1 [41].

Few studies have assessed ITGB2 in the context of obesity or pregnancy. However, various studies have suggested that ITGB2 is upregulated in chronic inflammatory conditions and could serve as a possible therapeutic target for diseases such as atherosclerosis [42, 43]. Nonetheless, there is emerging evidence that early-life inflammatory signaling promotes fat deposition and influences growth patterns [44].

### Clinical implications

Our finding that maternal serum PON3 is significantly and negatively associated with child weight SDS at 1 year of age adjusted for GA, but not birth weight, could point to the role of PON3 in programming fetal metabolic processes. While birth weight is more reflective of intrauterine conditions [45], postnatal growth is influenced not only by diet and birth weight, but also by lipid metabolism and oxidative stress [46]. We postulate that higher levels of PON3, an antioxidant, may reflect a protective maternal environment linked to lower infant weight gain.

Considering the inflammatory properties of ITGB2, our findings align with those of PON3. We suggest that higher ITGB2, a marker of immune activation, may indicate proinflammatory signaling that promotes increased postnatal weight. This could highlight a potential immune-metabolic axis influencing early growth trajectories.

### Research implications

As our cohort consisted of offspring born with appropriate-for-gestational-age birth weight, the associations of PON3 and ITGB2 with SGA or LGA birth weight or with early childhood obesity remain unknown. A better understanding of the roles of PON3 and ITGB2 in child growth could help identify mechanisms of fetal programming or even possible screening parameters for identifying offspring at risk for adverse developmental outcomes.

### Strengths and limitations

The strengths of this study include the longitudinal nature of observations, with follow-up data at 1 year of age available for 86 of the participants. We further provide an assessment of how biomarker trajectories evolve throughout pregnancy. To our knowledge, this is the first study showing significant associations between maternal biomarkers in late pregnancy and child weight at 1 year of age. Nonetheless, the small cohort size may limit the statistical power and generalizability of the results. Similarly, the cohort did not include pregnancies affected by SGA or LGA outcomes, further limiting the generalizability of our findings. Participants in this study were recruited at a single site in central Europe and were predominantly of Caucasian descent, which restricts the extrapolation of our results onto other populations with different socioethnical characteristics. Further, as Olink NPX values reflect relative protein expression and do not adjust for pregnancy-related plasma volume expansion, residual effects of hemodilution cannot be excluded, although this limitation applies to most circulating biomarker studies in pregnancy. It is also important to note that this study was based on a targeted proteomics approach restricted to the cardiovascular biomarkers included in the Olink Cardiovascular III panel; thus, proteins from other biological pathways that may influence fetal growth and neonatal outcomes were not assessed. Lastly, multivariable regression in small cohorts may lead to overfitting and thus inflate the results of variable effects [47].

### Conclusions

In conclusion, we found that the maternal serum biomarkers PON3 and ITGB2 during the third trimester of pregnancy are significantly and independently correlated with children's weight development during their first year of life. Importantly, most of these biomarkers did not correlate significantly and independently with the weight at birth.

Further studies are needed to understand the mechanisms in which ITGB2 and PON3 can directly affect weight gain and metabolism. Moreover, studies assessing cardiovascular biomarkers in pregnancies resulting in SGA offspring as well as LGA offspring are needed to determine whether these biomarkers also relate to fetal growth extremes and to clarify their potential role in long-term cardiovascular and metabolic health.

## Data Availability

Restrictions apply to the availability of some or all data generated or analyzed during this study to preserve patient confidentiality. The corresponding author will on request detail the restrictions and any conditions under which access to some data may be provided.
